# Supplementary material for: Subcellular second messenger networks drive distinct repellent-induced axon behaviors
Source: Nat Commun. 2023 Jun 27;14:3809. doi: 10.1038/s41467-023-39516-z (PMC10300027; doi:10.1038/s41467-023-39516-z)
Supplement: Supplementary file 1 — Reporting Summary [file 41467_2023_39516_MOESM1_ESM.pdf]

nature portfolio

Corresponding author(s): Xavier Nicol

Last updated by author(s): May 25, 2023

Reporting Summary

Nature Portfolio wishes to improve the reproducibility of the work that we publish. This form provides structure for consistency and transparency in reporting. For further information on Nature Portfolio policies, see our Editorial Policies and the Editorial Policy Checklist.

Statistics

For all statistical analyses, confirm that the following items are present in the figure legend, table legend, main text, or Methods section.

n/a	Confirmed	
☐	☒	The exact sample size (*n*) for each experimental group/condition, given as a discrete number and unit of measurement
☐	☒	A statement on whether measurements were taken from distinct samples or whether the same sample was measured repeatedly
☐	☒	The statistical test(s) used AND whether they are one- or two-sided<br>*Only common tests should be described solely by name; describe more complex techniques in the Methods section.*
☒	☐	A description of all covariates tested
☐	☒	A description of any assumptions or corrections, such as tests of normality and adjustment for multiple comparisons
☐	☒	A full description of the statistical parameters including central tendency (e.g. means) or other basic estimates (e.g. regression coefficient) AND variation (e.g. standard deviation) or associated estimates of uncertainty (e.g. confidence intervals)
☐	☒	For null hypothesis testing, the test statistic (e.g. *F*, *t*, *r*) with confidence intervals, effect sizes, degrees of freedom and *P* value noted<br>*Give P values as exact values whenever suitable.*
☒	☐	For Bayesian analysis, information on the choice of priors and Markov chain Monte Carlo settings
☒	☐	For hierarchical and complex designs, identification of the appropriate level for tests and full reporting of outcomes
☒	☐	Estimates of effect sizes (e.g. Cohen's *d*, Pearson's *r*), indicating how they were calculated

Our web collection on statistics for biologists contains articles on many of the points above.

Software and code

Policy information about availability of computer code

Data collection	No custom algorithm or software were used for data collection
Data analysis	No custom algorithm or software were used for data analysis

For manuscripts utilizing custom algorithms or software that are central to the research but not yet described in published literature, software must be made available to editors and reviewers. We strongly encourage code deposition in a community repository (e.g. GitHub). See the Nature Portfolio guidelines for submitting code & software for further information.

Data

Policy information about availability of data

All manuscripts must include a data availability statement. This statement should provide the following information, where applicable:

- Accession codes, unique identifiers, or web links for publicly available datasets
- A description of any restrictions on data availability
- For clinical datasets or third party data, please ensure that the statement adheres to our policy

The data that support this study are available within the Article, Supplementary Information or Source Data File

Human research participants

Policy information about studies involving human research participants and Sex and Gender in Research.

Reporting on sex and gender	n/a
Population characteristics	n/a
Recruitment	n/a
Ethics oversight	n/a

Note that full information on the approval of the study protocol must also be provided in the manuscript.

Field-specific reporting

Please select the one below that is the best fit for your research. If you are not sure, read the appropriate sections before making your selection.

☒ Life sciences ☐ Behavioural & social sciences ☐ Ecological, evolutionary & environmental sciences

For a reference copy of the document with all sections, see nature.com/documents/nr-reporting-summary-flat.pdf

Life sciences study design

All studies must disclose on these points even when the disclosure is negative.

Sample size	No sample size was performed. Statistical power computed a posteriori is provided.
Data exclusions	No data were excluded from the analysis, except for FRET imaging for which axons lacking a NO- ionomycin- or Forskolin-induced change in FRET reflecting an elevation of cGMP, Ca2+ or cGMP, respectively, were excluded from the analysis.
Replication	Replication data are provided in the Source Data file
Randomization	No active randomization was used. However the use of full litters led to randomize the allocation of the samples.
Blinding	Collapse assay: Blinding was performed on a few experiments and confirmed the results obtained on non-blinded datasets. For Live imaging experiments, blinding was not performed to make sure that no bias was introduced, with respect to the number of hours spent in vitro that might influence the behavior of developing axons.

Reporting for specific materials, systems and methods

We require information from authors about some types of materials, experimental systems and methods used in many studies. Here, indicate whether each material, system or method listed is relevant to your study. If you are not sure if a list item applies to your research, read the appropriate section before selecting a response.

Materials & experimental systems

n/a	Involved in the study	
☐	☒	Antibodies
☒	☐	Eukaryotic cell lines
☒	☐	Palaeontology and archaeology
☐	☒	Animals and other organisms
☒	☐	Clinical data
☒	☐	Dual use research of concern

Methods

n/a	Involved in the study	
☒	☐	ChIP-seq
☒	☐	Flow cytometry
☒	☐	MRI-based neuroimaging

Antibodies

Antibodies used	All antibody used are describe in the method section
Validation	Antibodies have been validated by the providers and checked in previous studies.<br>- Averaimo et al. 2016, Nat Commun (2016):<br>Western blots: GFP (Life Technologies), β-Adaptin (Santa Cruz), Caveolin (BD Transduction Laboratories)

	Immunocytochemistry on retinal growth cone in vitro: DsRed (Takara Bio), βIII-tubulin (Biolegend)<br>Whole mount immunostaining: DsRed (Takara Bio)<br>- Belle et al., Cell Rep (2014) :<br>Whole mount immunostaining: TAG1 (R&D Systems)

Animals and other research organisms

Policy information about studies involving animals; ARRIVE guidelines recommended for reporting animal research, and Sex and Gender in Research

Laboratory animals	Mated C57BL6/J and RjOrl:SWISS mice were purchased from Janvier Labs. Animals were housed on a 12 h light/12 h dark cycle in temperature- and humidity-controlled environment (19-23°C, 45-60%).<br>The culture of retinal explant and in utero electroporation were performed using E14.5 embryos. In utero electroporated animals were perfused at E18.5 and P15 to image the chiasm and superior colliculus, respectively.
Wild animals	No wild animals were used in this study
Reporting on sex	Embryos from dated matings (developmental stage stated in each section describing individual experiments) were not sexed during the experiments and the female over male ratio is expected to be close to 1.
Field-collected samples	No field-collected samples were used in this study
Ethics oversight	All animal procedures were performed in accordance with institutional guidelines and approved by the local ethics committee (C2EA-05: Comité d’éthique en expérimentation animale Charles Darwin; protocol APAFIS#22331-2019100814127972v4).

Note that full information on the approval of the study protocol must also be provided in the manuscript.

March 2021
