# Supplementary material for: Subcellular second messenger networks drive distinct repellent-induced axon behaviors
Source: Nat Commun. 2023 Jun 27;14:3809. doi: 10.1038/s41467-023-39516-z (PMC10300027; doi:10.1038/s41467-023-39516-z)
Supplement: Supplementary file 2 — Supplementary Information [file 41467_2023_39516_MOESM2_ESM.pdf]

SUPPLEMENTARY FIGURES

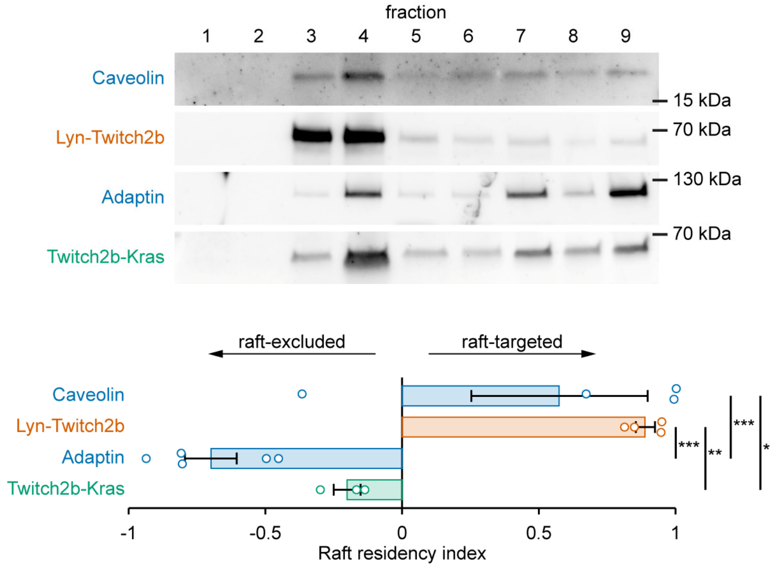

Supplementary Figure 1. Subcellular targeting of the Ca^{2+} biosensor Twitch2b.
The FRET Ca^{2+} biosensor Twitch2b was fused to a raft targeting- (Lyn) or a raft excluding-sequence (Kras) and electroporated in the developing retina. Membrane fractionation was performed to evaluate the subcellular localization of each construct. Lyn-Twitch2b is found in the same fraction as the raft-targeted protein Caveolin 1, whereas the pattern of Twitch2b-Kras mimics the distribution of Adaptin, a raft-excluded marker. The residency to lipid raft was quantified using a raft-targeting index calculated as $(I_{fraction\ 3} - I_{fraction\ 9})/(I_{fraction\ 3} + I_{fraction\ 9})$, where $I_{fraction\ 3}$ and $I_{fraction\ 9}$ are the fraction of the signal found in fraction 3 and 9, respectively. Thus, this index ranges from -1 (lipid raft exclusion) to 1 (lipid raft targeting). The mean, s.e.m. and individual data points are shown. * $P<0.05$; ** $P<0.01$; *** $P<0.001$; One-way ANOVA followed by Dunnett's post-hoc test. Source data, number of replicates and P values are provided as a Source Data file.

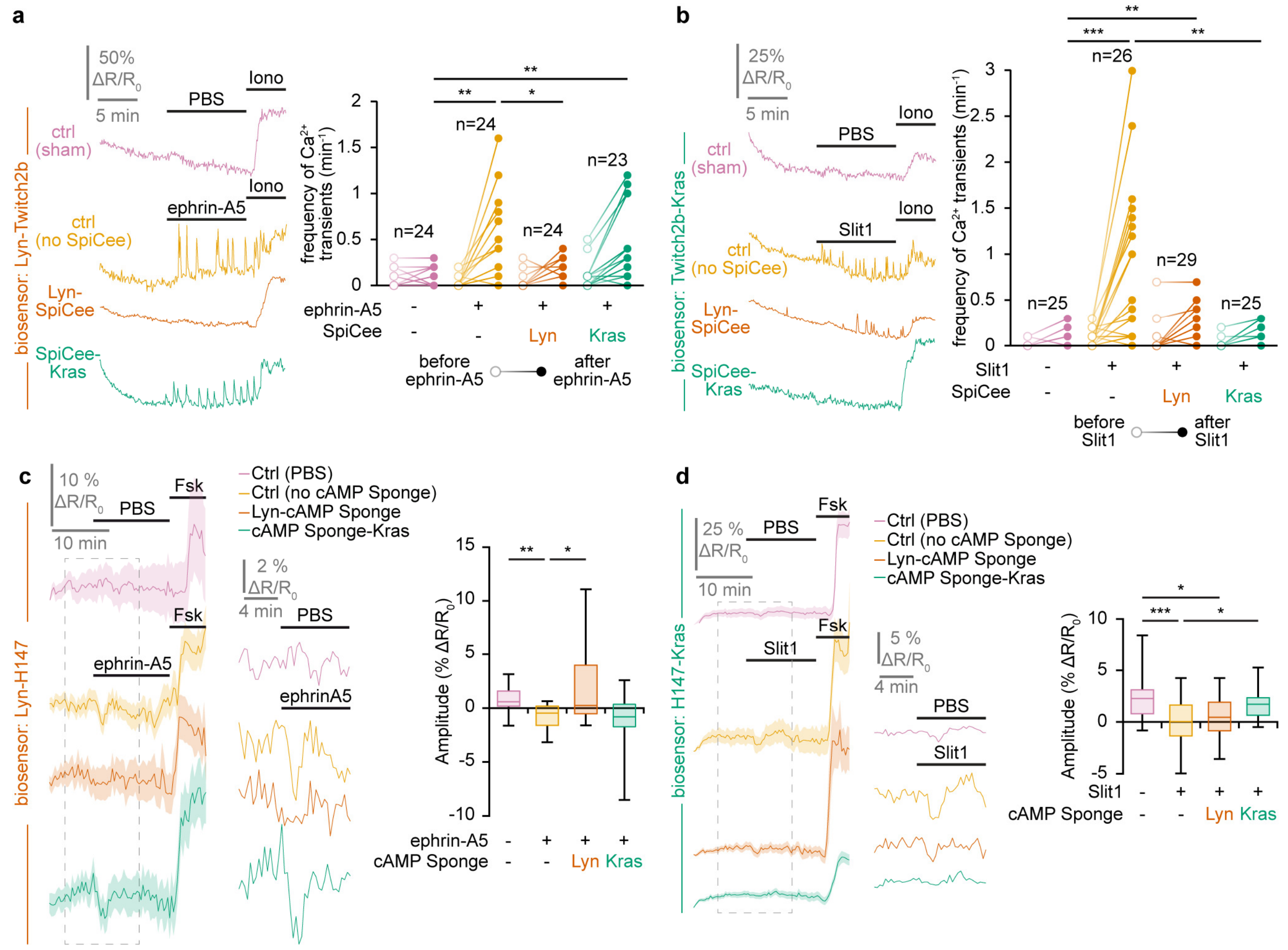

Supplementary Figure 2. Specificity of the subcellular buffering of second messengers.

(a) The lipid raft-targeted Ca^{2+} biosensor Lyn-Twitch2b was expressed in retinal explants alone or in combination with the Ca^{2+} scavengers targeted to (Lyn-SpiCee) or excluded from lipid rafts (SpiCee-Kras) and retinal axons were exposed to ephrin-A5. The ephrin-A5-induced and lipid raft-restricted elevation of Ca^{2+} transient frequency is abolished by the lipid raft-restricted Ca^{2+} scavenger Lyn-SpiCee, but not by its lipid raft-excluded equivalent SpiCee-Kras. An ionomycin (iono) stimulation leading to a Ca^{2+} elevation was achieved at the end of each recording to verify the functionality of the biosensor and the viability of the axon. Representative traces are shown. Individual data points are shown. * $P<0.05$; ** $P<0.01$; Kruskal-Wallis test followed by Mann-Whitney post-hoc tests. The number of quantified axons is indicated on the graphs.

(b) The lipid raft-excluded Ca^{2+} biosensor Twitch2b-Kras was expressed in retinal explants alone or in combination with the Ca^{2+} scavengers targeted to (Lyn-SpiCee) or excluded from lipid rafts (SpiCee-Kras) and retinal axons were exposed to Slit1. The Slit1-induced and lipid raft-excluded elevation of Ca^{2+} transient frequency is abolished by the lipid raft-excluded Ca^{2+} scavenger SpiCee-Kras, but not by its lipid raft-targeted equivalent Lyn-SpiCee. An ionomycin (iono) stimulation leading to a Ca^{2+} elevation was achieved at the end of each recording to verify the functionality of the biosensor and the viability of the axon. Representative traces are shown. Individual data points are shown. ** $P<0.01$; *** $P<0.001$; Kruskal-Wallis test followed by Mann-Whitney post-hoc tests. The number of quantified axons is indicated on the graphs.

(c) The lipid raft-targeted cAMP biosensor Lyn-H147 was expressed in retinal explants alone or in combination with the cAMP scavengers targeted to (Lyn-cAMP Sponge) or excluded from lipid rafts (cAMP Sponge-Kras) and retinal axons were exposed to ephrin-A5. The ephrin-A5-induced and lipid raft-restricted reduction in cAMP concentration is abolished by the lipid raft-targeted cAMP buffer Lyn-cAMP Sponge, but not by its lipid raft-excluded equivalent cAMP Sponge-Kras. A forskolin (Fsk) stimulation leading to a cAMP elevation was achieved at the end of each recording to verify the

functionality of the biosensor and the viability of the axon. The portion of the left traces enclosed in the dashed rectangle is shown magnified in the right part of the panel. Traces: mean ± s.e.m. Box-and-whisker plot elements: median, upper and lower quartiles, 10th and 90th percentiles. * $P<0.05$; ** $P<0.01$; Kruskal-Wallis test followed by Mann-Whitney post-hoc tests.

(d) The lipid raft-excluded cAMP biosensor H147-Kras was expressed in retinal explants alone or in combination with the cAMP scavengers targeted to (Lyn-cAMP Sponge) or excluded from lipid rafts (cAMP Sponge-Kras) and retinal axons were exposed to Slit1. The Slit1-induced and lipid raft-excluded reduction in cAMP concentration is abolished by the lipid raft-excluded cAMP buffer cAMP Sponge-Kras, but not by its lipid raft-targeted equivalent Lyn-cAMP Sponge. A forskolin (Fsk) stimulation leading to a cAMP elevation was achieved at the end of each recording to verify the functionality of the biosensor and the viability of the axon. The portion of the left traces enclosed in the dashed rectangle is shown magnified in the right part of the panel. Traces: mean ± s.e.m. Box-and-whisker plot elements: median, upper and lower quartiles, 10th and 90th percentiles. * $P<0.05$; *** $P<0.001$; Kruskal-Wallis test followed by Mann-Whitney post-hoc tests. Source data, number of replicates and P values are provided as a Source Data file.

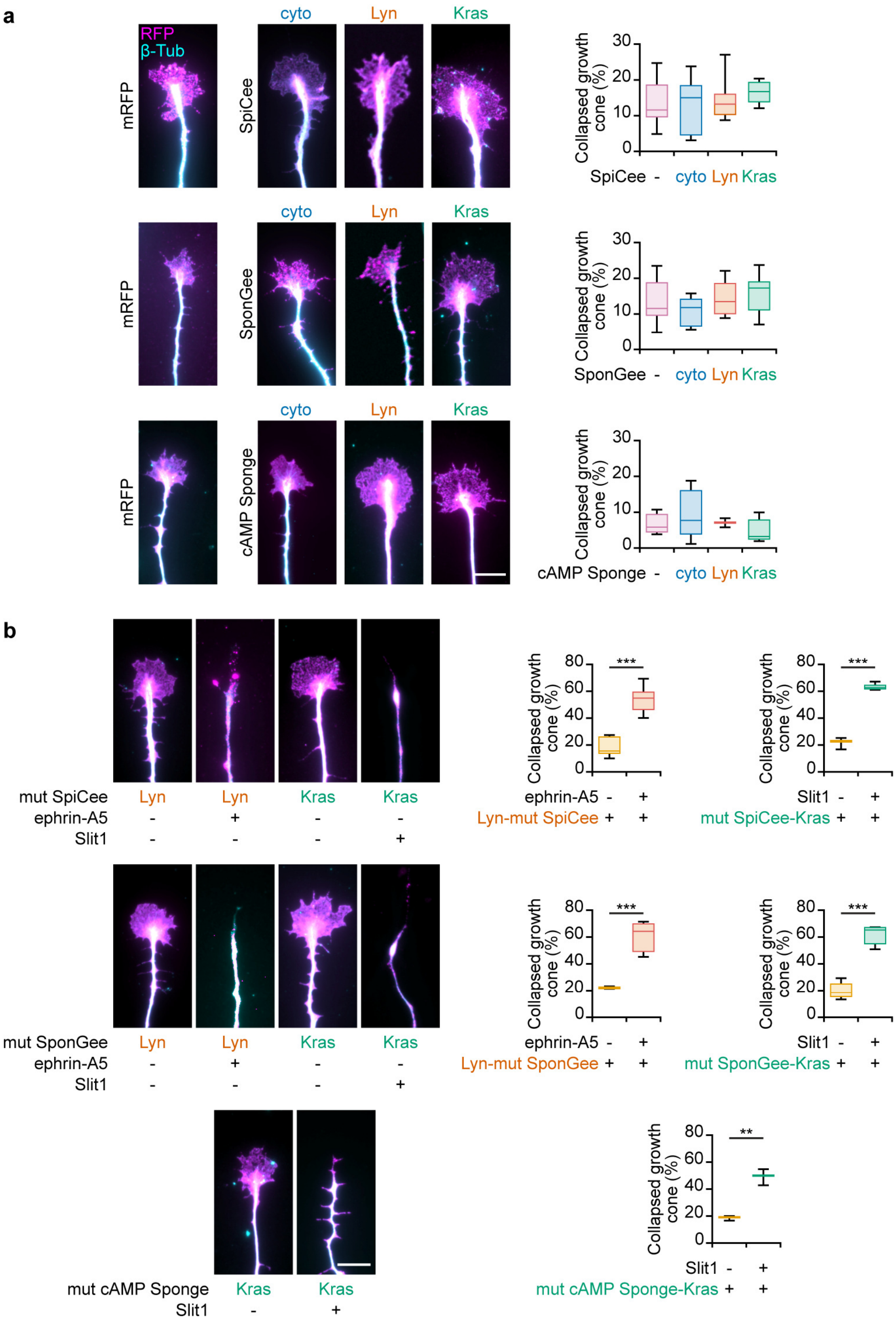

Supplementary Figure 3. The morphology of growing axons is not affected by SpiCee, SponGee or cAMP Sponge expression and the second messenger binding sites of these scavengers are required for them to alter the collapse of RGC growth cones.

(a) Retinal axons expressing SpiCee, SponGee or cAMP Sponge either lacking a targeting sequence (cyto), targeted to lipid rafts (Lyn) or excluded from this subcellular compartment (Kras) exhibit a morphology similar to mRFP-expressing axons. In particular, the number of collapsed growth cones is similar in all these experimental conditions. No statistically significant difference was found; one-way ANOVA followed by Dunnett’s post-hoc test.

(b) SpiCee, SponGee and cAMP Sponge carrying point mutations that abolish their ability to bind their targeted second messenger (mut SpiCee, mut SponGee and mut cAMP Sponge, respectively) do not

affect the ephrin-A5- or Slit1-induced collapse of retinal growth cones. ** $P<0.01$; *** $P<0.001$; two-tailed Student's t test.

Axons were immunolabeled with a βIII-tubulin and a Ds-Red antibody. The latter reports the expression of SponGee, SpiCee, cAMP Sponge, or their targeted or mutated variants. Scale bar, 10 µm. Box-and-whisker plot elements: median, upper and lower quartiles, 10th and 90th percentiles. Source data, number of replicates and P values are provided as a Source Data file.

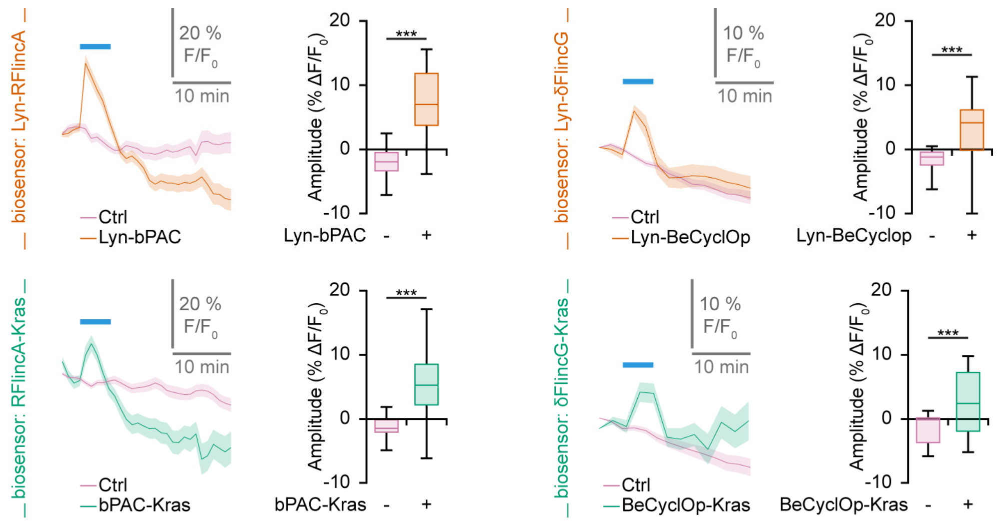

Supplementary Figure 4. Light exposure of subcellular targeted bPAC and BeCyclOP enables to impose changes in the concentration of cAMP and cGMP, respectively.

Light pulses (blue bar) induce an elevation of cAMP in Lyn-bPAC- (top left) and bPAC-Kras-expressing (bottom left) axons as compared to their respective controls exposed to light but that do not express Lyn-bPAC or bPAC-Kras. This elevation was detected by the subcellular-targeted biosensors Lyn-RFlincA and RFlincA-Kras, respectively. Of note, the cAMP concentration drops below the resting concentration after the release of bPAC simulation. Similarly, light exposure induces an elevation of cGMP in Lyn-BeCyclOp- (top right) and BeCyclOp-Kras-expressing (bottom right) axons as compared to their respective controls exposed to light but that do not express Lyn-BeCyclOp or BeCyclOp-Kras. This elevation was detected by the subcellular-targeted biosensors Lyn-δFlincG and δFlincG-Kras, respectively. Traces: mean ± s.e.m. Box-and-whisker plot elements: median, upper and lower quartiles, 10th and 90th percentiles. *** P<0.001; two-tailed Mann-Whitney test. Source data, number of replicates and P values are provided as a Source Data file.

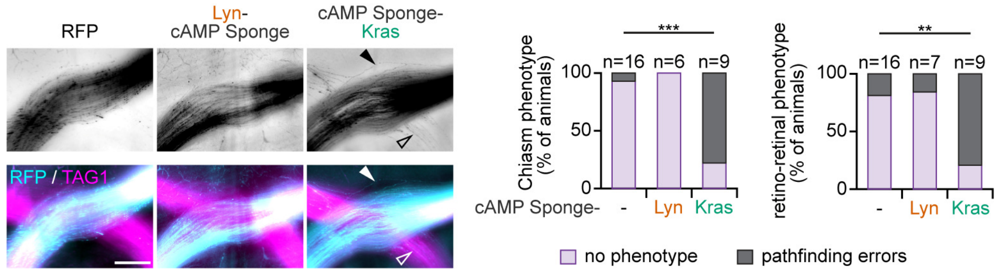

Supplementary Figure 5. Lipid raft-excluded scavenging of cAMP lead to misguided retinal at the optic chiasm.

cAMP Sponge-Kras-expressing axons exit the optic chiasm labeled with TAG1, by contrast to the axons of mRFP- and Lyn-cAMP Sponge-electroporated RGCs. An excess of retino-retinal axons is also detected in cAMP Sponge-Kras-electroporated animals, as compared to mRFP- and Lyn-cAMP Sponge-electroporated RGCs. The top row highlights the mRFP channels in which electroporated axons are seen. Closed arrowheads, axons exiting the optic chiasm; open arrowheads, retino-retinal axons. Scale bar, 200 µm. ** P<0.01; *** P<0.001; χ^2 test followed by χ^2 post-hoc tests. Source data, number of replicates and P values are provided as a Source Data file.
