# Supplementary material for: Subcellular second messenger networks drive distinct repellent-induced axon behaviors
Source: Nat Commun. 2023 Jun 27;14:3809. doi: 10.1038/s41467-023-39516-z (PMC10300027; doi:10.1038/s41467-023-39516-z)
Supplement: Supplementary file 4 — Description of additional supplementary files [file 41467_2023_39516_MOESM4_ESM.pdf]

Description of additional supplementary files

Supplementary Movie 1 : EphrinA5 and Slit1 induce distinct morphological changes of axonal growth cones in vitro. The growth of axons exposed to PBS was not affected (left video). EphrinA5 induced a growth cone collapse followed by a prompt retraction (middle video). Axons exposed to Slit1 exhibited a collapse of the growth cones but in contrast to axons encountering ephrin-A5, do not retract within the 20 minutes recorded (right video).
